# Factors associated with disordered gambling in Finland

**DOI:** 10.1186/1747-597X-8-24

**Published:** 2013-07-01

**Authors:** Sari Castrén, Syaron Basnet, Anne H Salonen, Maiju Pankakoski, Jenni-Emilia Ronkainen, Hannu Alho, Tuuli Lahti

**Affiliations:** 1 National Institute for Health and Welfare, Department of Mental Health and Substance Abuse Services, P.O. Box 30 Helsinki, FO 00271 Finland; 2 Institute of Behavioural Sciences, University of Helsinki, Faculty of Behavioural Sciences, Helsinki, Finland; 3 Institute of Clinical Medicine, Department of Medicine, Research Unit of Substance Abuse Medicine, University of Helsinki, Helsinki, Finland; 4 Department of Behavioural Sciences and Philosophy, University of Turku, Faculty of Social Sciences, Turku, Finland

**Keywords:** Disordered gambling, Pathological gambling, Population survey, Problem gambling, Public health, South Oaks Gambling Screen

## Abstract

**Background:**

The purpose of this study was to compare the socio-demographic characteristics of non-problem gamblers, problem gamblers and pathological gamblers, to investigate the association between gambling related factors and perceived health and well-being among the three subgroups of gamblers, and to analyse simultaneously socio-demographic characteristics, gambling related factors and perceived health and well-being and the severity of disordered gambling (problem gamblers and pathological gamblers).

**Methods:**

The data were collected through a nationwide telephone survey in 2011. Participants were selected through a random population sample of 15-74-year-old Finns. From that sample, persons with any past-year gambling involvement (N = 3451) were selected for a subsample for the descriptive and inferential analysis in the present paper. Gambling was assessed using the South Oaks Gambling Screen. Statistical significance was determined by chi-squared tests. The odds ratio and effect size were computed by using multivariate-adjusted multinomial logistic regression analysis.

**Results:**

The most significant socio-demographic characteristics (male gender, young age, education ≤12 years), gambling related factors (slot machine gambling, internet gambling) and perceived health and well-being (feeling lonely, smoking daily, risky alcohol consumption, mental health problems) explained 22.9 per cent of the variation in the severity of disordered gambling.

**Conclusion:**

Male gender and loneliness were found to be associated with problem gambling in particular, along with smoking and risky alcohol consumption. Mental health problems and risky alcohol consumption were associated with pathological gambling. These identified associations between disordered gambling, mental health problems and risky alcohol consumption should be taken into consideration when implementing screenings of disordered gambling.

## Introduction

Throughout the history people have been gambling. However, with the recent expansion of opportunities to gamble, gambling has become more problematic [1]. Today gamblers have the possibility to gamble more often and more frequently than ever before. Thus disordered gambling (DG) has become a serious public health concern worldwide. Most individuals gamble without any negative consequences due to gambling, but often excessive gambling leads to several adverse consequences to the gamblers, their significant others and to their communities [2]. The social and economic costs of DG are multitudinous. For example, the annual social cost of DG in the US is estimated to be 5 billion dollars [3,4].

The most severe pattern of DG is pathological gambling (PG) which is categorized by the Diagnostic and Statistical Manual of Mental Disorders (DSM-IV) as a disorder of impulse control [5]. PG meets at least five of the ten criteria listed in DSM-IV. In addition to PG, DSM-IV can also be used to identify a milder form of disordered gambling, problem gambling. Problem gambling meets 3–4 of the ten criteria listed in DSM-IV. Epidemiological studies estimate that the prevalence of PG is between 1.1% and 5.3% among the adult population [6-9]. Recent analysis by Williams and colleagues [10] stated that the standardized past-year prevalence of PG varied from 0.5% to 7.6% internationally. Currently in Finland the past-year prevalence of PG is estimated to be 1%, and problem gambling 1.7% [11].

DG and its consequences are often hidden, complex, multifaceted and multidimensional phenomena [12]. The individuals with particular socio-demographic characteristics seem to be at risk for the development of DG. For example male gender, young age, low socio-economic status, low educational level, divorced or single marital status, and in some studies, a minority status [3,13-16] have been linked to an increased risk for DG. Psychiatric comorbid illnesses have also been recognized to be common amongst persons suffering from DG [17-19]. For example, severe depression, mood disorders, the use of nicotine and alcohol use disorder have been associated with DG [20]. Along with specific socio-demographic characteristics and psychiatric comorbid illnesses, availability of gambling venues is also associated with the prevalence of gambling [15,21,22]. These associations have been studied broadly worldwide. However, amount of studies from Finland are so far rather limited [11]. Therefore, more information is needed in order to develop and establish effective methods for prevention and treatment of DG in a Finnish cultural-context. Our research establishes new information as only few studies have earlier examined simultaneously socio-demographic characteristics, gambling related factors, as well as perceived health and well-being [9].

Given the rapid growth and the increasing availability of gambling in varying frequencies and forms at the present, gambling has become more accessible worldwide [8,23,24], both internationally and in Finland. In Finland especially with a large amount of slot machines available as well as with a growing availability of unregulated internet sites worldwide. As excessive gambling has the potential to become disordered, it is important to understand which groups have elevated risk to develop DG. Therefore, in this study we first compare the socio-demographic characteristics (gender, age, education, marital status) of non-problem gamblers, problem gamblers and PG’s. Second, we investigate the association between gambling related factors (onset age, problem gambler close by, gambling frequency, money gambled and type of gambling) among the subgroups of gamblers. Third, we investigate the association between perceived health and well-being (loneliness, smoking, alcohol consumption, mental health and general health) among the subgroups of gamblers. Fourth, we analyse simultaneously socio-demographic characteristics, gambling related factors and perceived health and well-being and the severity of DG. These analyses are necessary for to find out the possible vulnerability factors related to DG and to develop early screenings of DG.

## Methods

This study is based on a cross-sectional nationwide telephone survey entitled the Finnish Gambling 2011 [11]. The data were collected between 3rd October 2011 and 14th January 2012. Participants were selected from the Finnish Population Register by using a random sample of 15-74-year-old Finns. The sample size was 16,000, of whom 11,129 had a registered telephone number. Before the telephone interview, the participants received an introductory letter describing the purpose of the study. The participants, whose phone number was not in the Finnish Population Register, were sent a letter requesting their willingness to participate in the survey. Eventually a total of 4,484 participants completed the study. From that sample, participants with any past-year gambling involvement (N = 3,451) were drawn for this study. The sampling weights based on age, gender and residency of the Finnish population [11] were applied to all descriptive and inferential analysis. The ethics committee of the National Institute for Health and Welfare approved the research protocol.

### Measurements

#### Gambling behaviour

Gambling behaviour was measured by using a 12-month time frame with the South Oaks Gambling Screen (SOGS) originally developed by Lesieur and Blume [24], with a total score of 20. SOGS is scored as: 0–2 = non-problem gamblers, 3–4 = problem gamblers, ≥5 = probable pathological gamblers. The Cronbach alpha for the SOGS was 0.913.

#### Socio-demographic characteristics

Socio-demographic characteristics analysed in this study included gender, age, education and marital status (Table 1).

**Table 1:** Association between socio-demographic characteristics among the subgroups of gamblers

	**All gamblers**	**Non-problem gamblers**	**Problem gamblers**	**Pathological gamblers**	**X**^**2 **^**test**
	**N = 3451**	**n = 3345**	**n = 67**	**n = 39**	
**Characteristics**	**%**	**%**	**%**	**%**	
Gender					
Male	53.2	52.2	85.7	70.0	X^2^ = 35.374, df = 2, p ≤ 0.001
Female	46.8	47.8	14.3	30.0	
Age, in years					
15-24	14.2	13.9	21.7	25.6	
25-34	18.2	17.9	23.2	28.2	X^2^ = 15.061, df = 6, p = 0.019
35-49	26.9	27.0	26.1	15.4	
≥ 50	40.7	41.1	29.0	30.8	
Education					
≤ 12 years education	40.0	39.5	57.1	47.5	X^2^ = 9.792, df = 2, p = 0.007
> 12 years education	60.0	60.5	42.9	52.5	
Marital status					
Married/registered relationship	48.1	48.9	27.9	25.0	X^2^ = 31.040, df = 6, p ≤ 0.001
Cohabiting	17.9	18.0	11.8	25.0	
Separated/divorced/widowed	10.2	10.0	16.2	15.0	
Single	23.7	23.1	44.1	35.0	

Significance (p) is determined by chi-squared (X^2^) test; The data were weighted based on gender, age and residency.

#### Gambling related factors

Gambling related factors were onset age, problem gambler (significant other) gambling frequency, gambling expenditure and the types of games gambled (Table 2). The following questions were used with each factor: a) onset age, with the question of, ‘When did you start gambling?’ as a continuous factor; b) gambling of significant others (father, mother, sister or brother, grandparent, spouse, child, close friend) with the question of ‘Do any of the following people have or have had problems with gambling?’ with answering options (yes, no, do not know). This was a categorical factor, that was recorded into a dichotomous factor; c) gambling frequency, which was recoded into two categories (once a week or more/rarely than weekly); d) gambling expenditure, with the question of ‘How much money did you spend into gambling during the past week?’. This was a continuous factor, that was recoded into three categories (do not know, 0-5€, 5€ or more); e) type of gambling with five options: lotto, scratch cards, slot machines, casino gambling or gambling in the internet during the past 12 months, as a categorical factor.

**Table 2:** Association between gambling related factors and subgroups of gamblers

	**All gamblers**	**Non-problem gamblers**	**Problem gamblers**	**Pathological gamblers**	**X**^**2 **^**test**
	**N = 3451**	**n = 3345**	**n = 67**	**n = 39**	
**Gambling related factors**	%	**%**	**%**	**%**	
Onset age, years					X^2^ = 22.174, df = 2, p ≤ 0.001
< 18	56.6	55.8	75.7	82.5	
≥ 18	43.4	42.2	24.3	17.5	
Problem gambler (significant other)					X^2^ = 33,177, df = 2, p ≤ 0.001
Yes, at least one	19.9	19.2	37.1	47.5	
No, none	80.1	80.8	62.9	52.5	
Gambling frequency					
Once a week or more	45.8	44.4	88.4	77.5	X^2^ = 69.094, df = 2, p ≤ 0.001
Rarely than weekly	54.2	55.6	11.6	22.5	
Money gambled, past week					
Do not know	19.6	19.5	21.4	25.6	
0-5 euro	50.8	52.2	10.0	17.9	X^2^ = 80.405, df = 4, p ≤ 0.001
> 5 euro	29.5	28.3	68.6	56.5	
Played lotto, past 12 months					
Yes	87.5	87.6	87.1	80.0	X^2^ = 2.112, df = 2, p = 0.348
No	12.4	12.4	12.9	20.0	
Played scratch cards, past 12 months					
Yes	44.0	43.4	62.3	62.5	X^2^ = 15.451, df = 2, p ≤ 0.001
No	56.0	56.6	37.7	37.5	
Played slot machines, past 12 months					
Yes	42.4	40.7	90.0	82.5	X^2^ = 94.750, df = 2, p ≤ 0.001
No	57.6	59.3	10.0	17.5	
Played casino, past 12 months					
Yes	2.8	2.4	7.2	30.8	X^2^ = 117.664, df = 2,p ≤ 0.001^†^
No	97.2	97.6	92.8	69.2	
Internet gambling, past 12 months					
Yes	24.5	23.6	48.6	55.0	X^2^ = 43.377, df = 2, p ≤ 0.001
No	75.5	76.4	51.4	45.0	

Significance (p) is determined by chi-squared (X^2^) test; ^†^33.3% cells have expected count less than 5; the data were weighted based on gender, age and residency.

#### Perceived health and well-being

Factors related to health and well-being included gamblers’ perceptions of loneliness, daily smoking, risky alcohol consumption, mental health and general health (Table 3). Loneliness was measured by using a question ‘Do you feel lonely?’ with five options, which were recoded into two categories (all the time/often and sometimes/rarely/never). Frequency of smoking was evaluated by using a question ‘Have you smoked during the past 12 months?’ with a 3-point Likert scale (daily, randomly, not at all). Random smokers and non-smokers were grouped into the same group for the analysis. Consumption of alcohol was measured by using the modified version of the Alcohol Use Disorders Identification Test, AUDIT-C [25]. AUDIT-C is a 3-item screen, which is used to identify those persons who are hazardous drinkers or have active alcohol use disorders (including alcohol abuse or dependence). The AUDIT-C is a 5-point Likert scale with scoring: a = 0 point, b = 1 point, c = 2 points, d = 3 points and e = 4 points. In this study, the total scores of AUDIT-C were counted by summing up the points for each item, and cut-off points recommended by Seppä [26] were used to define risky drinking among males (score ≥ 6) and females (score ≥5). The Cronbach alpha for the AUDIT-C was 0.611.

**Table 3:** Association between perceived health and well-being and subgroups of gamblers

	**All gamblers**	**Non-problem gamblers**	**Problem gamblers**	**Pathological gamblers**	**X**^**2 **^**test**
	**N = 3451**	**n = 3345**	**n = 67**	**n = 39**	
**Perceived health/well-being**	%	**%**	**%**	**%**	
Feeling lonely					X^2^ =27.509, df = 2, p ≤ 0.001
All the time/often	17.3	16.7	38.6	30.0	
Never/very rarely/sometimes	82.7	83.3	61.4	70.0	
Smoking					X^2^ =57.468, df = 2, p ≤ 0.001
Daily smoking	19.9	18.8	48.6	47.5	
Not at all/occasionally	80.1	81.2	51.4	52.5	
Alcohol risk consumer, AUDIT-C					
At risk	28.3	26.9	68.8	71.4	X^2^ =86.394, df = 2, p ≤ 0.001
Not at risk	71.7	73.1	31.2	28.6	
Mental health, MHI-5					
Clinically significant problem	3.3	3.0	8.6	17.9	X^2^ =33.024, df = 2, p ≤ 0.001
No problem	96.7	97.0	91.4	82.1	
General health					
Bad/somewhat bad	2.8	2.7	4.3	7.7	X^2^ =17.159, df = 4, p = 0.005^†^
Average	13.0	12.8	27.1	10.3	
Good/somewhat good	84.2	84.5	68.6	82.0	

*AUDIT-C*, the Alcohol Use Disorders Identification Test, score for risk consumption ≥5 among women and ≥6 among men; *MHI-5*, the Mental Health Inventory, scale 1–100, clinically significant problem ≤52. Significance (p) is determined by chi-squared (X^2^) test; the data were weighted based on gender, age and residency; ^†^22,2% cells have expected count less than 5.

The mental health of the participants was assessed by using the Mental Health Inventory (MHI-5) [27] comprising the following five items: nervousness, blues, jollity, calmness and happiness. MHI-5 was measured by using a 6-point Likert scale scoring: 1 = all of the time, 2 = most of the time, 3 = a good bit of the time, 4 = some of the time, 5 = a little of the time, 6 = none of the time. The total scores of MHI-5 factors were calculated by summing up the score of each item and the sums (range 4–30) were scaled into 1–100. Cut-off score of 52 or less was used: lower scores indicate clinically significant mental health problems [28]. The Cronbach alpha for the MHI-5 was 0.768. General health was inquired by using a question ‘How is your general health at present?’, with five options recoded into three categories (bad/somewhat bad, average and good/somewhat good).

### Statistical analysis

The analyses were carried out in two steps. First, chi-square test was used to compare the statistical significance (p) of the associations of the categorical factors and the three subgroups of gamblers (Tables 1, 2 and 3). The factors for these bivariate analyses were chosen as based on strong evidence gained from the previous studies. All categorical factors are presented using frequencies and percentages.

Then factors associated with the severity of DG were explored using a multivariate-adjusted multinomial logistic regression analysis (multinomial regression analysis). In this analysis, problem gamblers and PG’s were compared with non-problem gamblers. Selected factors consisted of socio-demographic characteristics, gambling related factors and perceived health and well-being and they were included in the final model simultaneously (Table 4).

**Table 4:** Simultaneously analysed factors: socio-demographic characteristics, gambling related factors and perceived health and well-being and the severity of disordered gambling (Problem and Pathological gambling)

		**Problem gambling**^**a**^		**Pathological gambling**^**a**^	
		**n = 67**		**n = 39**	
	**Factor**	**OR**	**95% CI**	**OR**	**95% CI**
**Socio-demographic**					
	Male	2.48*	1.20–5.12	1.10	0.49–2.46
	15–34 years old	0.86	0.50–1.46	1.29	0.63–2.66
	≤12 years education	1.53	0.90–2.60	1.25	0.61–2.54
**Gambling related**					
	Played slot machines, past 12 months	6.88***	3.05–15.56	4.70**	1.72–12.85
	Internet gambling, past 12 months	2.15**	1.26–3.38	2.88**	1.40–5.92
**Perceived health and well-being**					
	Feeling lonely	3.47***	1.98–6.05	1.78	0.78–4.04
	Smoking daily	2.01*	1.15–3.49	1.58	0.74–3.37
	Risk alcohol, AUDIT-C	2.57**	1.43–4.63	3.09**	1.38–6.94
	Mental health problem, MHI-5	1.40	0.50–3.88	4.01**	1.41–11.43
**Nagelkerke = 0,229**					

^a^Reference group: Non-problem gamblers (n = 3345); The data (N = 3451) were weighted based on gender, age and residency; Multivariate-adjusted multinomial logistic regression analysis; * < 0.05, ** < 0.01, *** < 0.001; *AUDIT-C*, the Alcohol Use Disorders Identification Test, score for risk consumption ≥5 among women and ≥6 among men; *MHI-5*, the Mental Health Inventory, scaled into 1–100, clinically significant problem ≤52.

Socio-demographic characteristics (gender, age and education) used in the model carry strong theoretical evidence from past studies. To precisely optimise the model, two game types, which represent the most widespread accessibility and addictive potential, slot machine and internet gambling, were included into the model as gambling related factors. Finally, loneliness, daily smoking, risky alcohol consumption and overall mental health (MHI-5) represented significant factors related to perceived health and well-being.

The best fitting model was chosen by exploring different combinations of factors and comparing different models using the coefficient of determination (R squared). Results of the multinomial regression model are presented as odd ratios (OR) and their corresponding 95% confidence intervals (CI). Goodness of fit was assessed using the Nagelkerke’s R^2^.

## Results

### Bivariate analysis: associations between socio-demographic characteristics and the subgroups of gamblers

The socio-demographic characteristics of the different subgroups of gamblers are summarized in Table 1. There were 3,451 participants (53.2% males and 46.8% females) with the mean age of 44.27 years (SD = 15.97). Overall, there were a greater proportion of males than females in all of the subgroups of gamblers. Compared with non-problem gamblers (52.2%) the percentage of males was greater amongst problem gamblers (85.7%) and PG’s (70.0%), (χ2 = 35.374, *df =* 2, p <0.001). According to our results, PG’s were younger compared to the other subgroups of gamblers (χ2 = 15.061, *df =* 2, p <0.019). There were statistically significantly more gamblers with twelve or less years of education in the problem gambling group (57.1%) compared to non-problem gamblers (39.5%) and to PG’s (47.5%), (χ2 = 9.792, *df =* 2, p <0.007). Most of the non-problem gamblers (66.9%) were married or lived in a registered relationship or were cohabiting, while the corresponding figures for problem gamblers were 39.7% and for PG’s 50.0%.

### Bivariate analysis: associations between gambling related factors and the subgroups of gamblers

Association between gambling related factors and the subgroups of gamblers are presented in Table 2. Onset age of gambling, namely below 18 years, was lower among problem and PG’s than among non-problem gamblers (χ2 = 22.174, *df =* 2, p <0.001). Problem gamblers and PG’s had or have had a problem gambler (significant other) more often than the non-problem gamblers (χ2 = 33.177, *df =* 2, p <0.001). Problem gamblers (88.4%) gambled more frequently (once a week or more) as compared to PG’s (77.5%) or non-problem gamblers (44.4%).

Problem gamblers spent more money on gambling than the other subgroups of gamblers (more than 5€ per week). However, the percentage of gamblers who did not know the amount they had spent on gambling was the greatest among PG’s (χ2 = 80.405, *df =* 2, p <0.001).

Lotto was the most often gambled game among all subgroups of gamblers. Non-problem gamblers gambled lotto (87.6%) slightly more often than problem gamblers (87.1%) or PG’s (80.0%), (χ2 = 2.112, *df =* 2, p <0.348). Scratch cards were gambled more frequently by problem gamblers (62.3%) and PG’s (62.5%) as compared to non-problem gamblers (43.4%), (χ2 = 15.45, *df =* 2, p <0.001). Similarly, slot machine gambling was the most prevalent among problem gamblers: 90.0% of the problem gamblers, 82.5% of the PG’s and 40.7% of the non-problem gamblers (χ2 = 94.750, *df =* 2, p <0.001) gambled slot machines. Casino gambling was the most prevalent among PG’s (30.8%) as compared with problem gamblers (7.2%) or non-problem gamblers (2.4%), (χ2 = 117.664, *df =* 2, p <0.001). Internet gambling was also the most prevalent among PG’s (55%) as compared to problem gamblers (48.6%) and non-problem gamblers (23.6%).

### Bivariate analysis: Perceived health and well-being and the subgroups of gamblers

Associations between perceived health and well-being and the subgroups of gamblers are presented in Table 3. Problem gamblers reported feelings of loneliness more often than the other subgroups of gamblers (χ2 = 27.509, *df =* 2, p <0.001). Problem gamblers also smoked slightly more on a daily basis than other subgroups of gamblers (χ2 = 57.468, *df =* 2, p <0.001). According to our results PG’s consumed more alcohol in a risky level (71.4%) than problem gamblers (68.8%) and non-problem gamblers (26.9%), (χ2 = 86.394, *df =* 2, p <0.001). PG’s also experienced clinically significant mental health problems more often than the other subgroups of gamblers (χ2 = 33.024, *df =* 2, p <0.001). However, with general health, there were no significant differences between the studied subgroups of gamblers. All in all, problem gamblers reported loneliness and smoked tobacco more than PG’s. PG’s, in turn, consumed alcohol at a risky level and had mental health problems more often than problem gamblers.

### Multinomial regression analysis: simultaneously analysed factors and the severity of DG

The simultaneously analysed socio-demographic characteristics, gambling related factors and perceived health and well-being and the severity of DG was examined by multinomial regression analysis (Table 4). In this analysis, male gender was the only socio-demographic characteristic that was statistically significantly associated with problem gambling (OR 2.48, CI 1.20-5.12). Young age (15–35) and education ≤ 12 years were not significantly associated with either problem gambling or PG. Game type was significantly associated with DG. Past-year slot machine gambling was significantly associated with problem gambling (OR 6.88, CI 3.05-15.56) and PG (OR 4.70, CI 1.72-12.85). Likewise was the past-year internet gambling associated with problem gambling (OR 2.15, CI 1.26-3.38) and PG (OR 2.88, CI 1.40-5.92). Associations with perceived health and well-being, were found to be significant with problem gambling as follows: loneliness (OR 3.47, CI 1.98-6.05), daily tobacco smoking (OR 2.01, CI 1.15-3.49) and risky alcohol consumption (OR 2.57, CI 1.43-4.63). Similarly, risky alcohol consumption was associated with PG statistically significantly (OR 3.09, CI 1.38-6.94). In addition, mental health problems were significantly associated with PG (OR 4.01, CI 1.41-11.43).

In the multinomial model, socio-demographic characteristics (male gender, young age, education ≤ 12 years), gambling related factors (played slot machines, internet gambling) and perceived health and well-being (loneliness, daily tobacco smoking, risky alcohol consumption, mental health problems) explained 22.9% of the variation in the severity of DG.

## Discussion

### Socio-demographic characteristics

According to our bivariate analysis, socio-demographic characteristics e.g., male gender, low level of education, single marital status and young age were all associated with DG. Similarly, risky alcohol consumption, smoking and loneliness were all associated with problem gambling and more severe mental health problems with PG. These findings are in line with previous research [29-33].

Young males are characteristically more often sensation seekers and thus they have a higher vulnerability to develop addictions [34-36]. Also in the multinomial regression analysis, a strong association between male gender and severity of DG were found. Even though prevalence of DG is greater in males than females, the progression of DG is faster with females [37]. In our study 30% of the PG’s were females. It has been proposed that the main reasons for females to gamble are often boredom, loneliness and isolation. Thus females tend to seek less adventurous gambling types and choose games that maximize their gambling time [38,39] to offer an escape from feeling isolated and lonely.

In the bivariate analysis, there were significantly more young gamblers (ages 15–24 and 25–34) in both DG subgroups. On the contrary, in a multinomial regression analysis, the age was no longer as strongly associated with the severity of DG, nor was this relationship clearly linear. This can be explained by the inclusion of other explanatory factors in the model, such as other socio-demographic characteristics, as well as gambling and health-related variables.

### Gambling related factors

In the bivariate analysis, onset age of gambling was associated with both subgroups of DG, as stated earlier by Volberg et al. [40]. In addition, our results show problem gamblers to gamble more frequently and to spend more money on gambling on a weekly basis than PG’s. On the other hand, most of the PG’s did not know how much money they had spent on gambling. Not knowing how much one has spent on gambling may reflect the very nature of the gambling pathology: denial of the problem. According to Williams and Volberg [41,42] ‘being ahead or in a winning state’ may well reflect gamblers biased perception of a winning state. Biased perception means that wins are well remembered and maximized and losses are forgotten or minimized.

PG’s gambled more frequently both internet and casino games than problem gamblers. Problem gamblers in turn preferred slot machine gambling more compared to PG’s. This difference could be explained perhaps by PG’s chasing losses by larger bets as based on ‘gamblers fallacy’. In turn, problem gamblers, that do not meet the full criteria of PG, may have temptation to try their ‘luck’ with slot machines, perhaps due to their easy accessibility. Slot machine gambling has been reported as the most problem causing type of gambling in Finland [43-45]. In Finland, slot machines are openly scattered in shopping centres, small shops, kiosks, restaurants, casino and casual gambling arcades. This is why a concern has emerged especially towards slot machine gambling. Griffiths [46] classified slot machine gambling as having a high addictive potential due to its fast tempo and other properties. Besides the easy access and availability, slot machines are likely to increase involvement in gambling and the development of DG [47-49]. Casino, internet and slot machine gambling are all classified as addictive gambling types [50,51].

Both subgroups of DG gambled lotto rather frequently. In a Finnish context, lotto is the most popular game type in general. The addictiveness of lotto comes from the structural characteristics of the game. Lotto has the potential to be gambled at various intervals be it yearly, weekly or daily. The low cost chance of winning a very large jackpot prize urges gamblers to buy lotto repeatedly. However, the low event frequency of lotto may explain why other types of gambling are more addictive than lotto [52].

### Perceived health and well-being

Based on multinomial regression analysis**,** a new finding was that problem gamblers in particular, reported to be lonelier than non-problem gamblers or PG’s. PG’s, on the other hand, reported having more mental health problems than problem gamblers and non-problem gamblers. Loneliness, which was associated with problem gambling, can be seen as less severe than overall problems with mental health. Therefore, detecting loneliness among problem gamblers is important and could be used, for example, as a guiding tool/question in the screening of DG. Loneliness, analysed simultaneously with certain socio-demographic characteristics, such as being a young male, may lead to a more severe form of DG or more severe mental health problems if not tackled early enough. Loneliness [39] may be a result or consequence of gambling, given the fact that this is a cross-sectional design. Both loneliness and social isolation is associated with gambling especially among female gamblers [39]. Boredom, which refers to lack of interest in general, is also linked with gambling [53,54].

According to this study DG’s were smoking more and consuming alcohol at risk level more often than non-problem gamblers. This finding is in line with earlier studies: PG is a frequent comorbid diagnosis among substance abusers and vice versa [55]. In addition to substance abuse, also mental health problems such as depression co-occur at high rates amongst DG’s [55].

Our multinomial regression analysis shows that there are certain similarities amongst two subgroups of DG’s (e.g. type of games gambled and risky alcohol consumption) and therefore, it would be beneficial to use these identified factors in prevention and early detection programs as one additional guideline. DG may get worse over time if not detected early enough. Thus there is an urgent need to better identify and prevent DG before it becomes more difficult to overcome.

DG and its consequences are often hidden, due to feelings of shame and guilt related to excessive gambling and denial of the problem and therefore can be unrecognized for a considerable time. Thus the early identification of DG within the health care system is often difficult. However, by increasing general health practitioners’ awareness of the symptoms and most common comorbidities of DG, early detection and better screening of gambling problems can be increased. Based on our multinomial regression analysis, loneliness, smoking and risky alcohol consumption were found to be associated with problem gambling and should be taken into account when screening and treating DG.

### Relevance to public health perspective: prevention, protection and detection

Moreover, our study’s multinomial regression analysis found slot machine gambling to be associated with DG. Slot machine gambling is known to be a game of pure chance and therefore by offering accurate knowledge about the features of the games, such as the existence of erroneous beliefs related to gambling or probabilities of winning in different games, the harms caused by gambling can be reduced and treated [56-62]. Also a recent publication from the European Commission (EC) recommended more clear information to be given about gambling products [63]. The same has been stated in the Reno Model [64]. Therefore, all games, betting and lottery tickets should have a product warning providing information about the harmful effects and possible negative consequences of gambling. Moreover, anti-stigma campaigns could also increase the public knowledge about DG and could increase the treatment seeking level of the individuals with disordered gambling [9].

It is important to acknowledge that certain game types and their availability and easy accessibility are risk factors for the development of DG [44,65-67]. An alerting notion in two Finnish reports [41] is that of a new type of game that is offered via internet: internet slot machine games, where two addictive game types are combined with 24/7 accessibility. As a solution to these maintaining factors of gambling, Marshall [68] sets a good comparison on other public health issues, such as obesity, tobacco smoking and alcohol problems. In all of these public health issues the availability (e.g. fast food, cigarettes and alcohol) has worsened the condition at hand. Increasing the public awareness of the health issues involved, would give people more choice in their behaviour. This same approach is relevant and could also work with gambling issues.

Adams et al. [69] have brought up the question of how policy makers could respond to the harms caused by gambling. Harm minimization initiatives have been targeted into reducing availability and increasing education about harms of gambling. A good example from a harm minimization strategy comes from the Australian state of Victoria, where the number of Electronic Gambling Machines (EGM’s) was reduced particularly in low-income communities, where the gambling was linked with harmful gambling [68,70]. Increasing such health promotion programs to include harms of gambling is recommended. As it is, expansion of commercial gambling is taking place on a global basis, especially internet gambling, which provides round-the-clock access to various gambling types, increasing the number of people getting involved and perhaps encountering some form of DG.

### Limitations

First, a review of the population-based gambling studies indicates that the mean response rate in studies using telephone interview is generally 52.5% [10]. Therefore, the response rate of this study can be considered as low (39.9%). Moreover, the proportion of young male participants was lower than the national average [11]. This is a typical phenomenon in gambling studies and an important notion since the prevalence of DG is typically high among young males [14-16]. The data weighting was performed to correct this bias. Second, the total number of problem gamblers and PG’s was 106. However, international comparison indicates that this figure was higher than the international median of 52 [10]. Third, the SOGS instrument used in assessing gambling behaviour was originally developed in clinical context [24]. On the other hand, the version with a shorter time frame has been validated in a population-based study [71]. In this study, the internal consistency and reliability of the SOGS appeared to be good. Fourth, this study is limited by the cross-sectional study design; therefore, no conclusions about the causal connection can be done. At the same time, the multinomial method used enhances the reliability since it notices the effect of the simultaneously analysed factors.

## Conclusion

The consequences of gambling at societal, individual and familial level, calls for new actions both in Finland and internationally. Male gender and loneliness were found to be associated with problem gambling in particular, along with smoking and risky alcohol consumption. Mental health problems and risky alcohol consumption were associated with pathological gambling. These identified associations between disordered gambling, mental health problems and risky alcohol consumption should be taken into consideration when implementing more in-depth and targeted screening of DG. It is also important to consider these associations when planning treatment of DG.

## Competing interests

The authors declare that they have no competing interests.

## Authors’ contribution

The study design was develop by all authors. SC, SB, AHS, MP, HA, JER, TL have made substantial contributions to the analysis and interpretation of the data. SC, SB, AHS, MP, HA, TL have been involved in drafting the manuscript and revising it critically. All authors have given final approval of this version to be published.
